# Two new species of the bamboo-feeding genus *Bambusicaliscelis* Chen & Zhang, 2011 from China (Hemiptera, Fulgoromorpha, Caliscelidae)

**DOI:** 10.3897/zookeys.776.24355

**Published:** 2018-07-26

**Authors:** Nian Gong, Lin Yang, Xiang-Sheng Chen

**Affiliations:** 1 Institute of Entomology, Guizhou University, Guiyang, Guizhou University Guiyang China; 2 Guizhou, 550025, PR China Guizhou University Guiyang China; 3 The Provincial Special Key Laboratory for Development and Utilization of Insect Resources, Guizhou University, Guiyang, Guizhou, 550025, PR China Guizhou University Guiyang China

**Keywords:** Caliscelini, planthopper, taxonomy, bamboo, distribution

## Abstract

Two new species of the bamboo-feeding planthopper genus *Bambusicaliscelis* Chen & Zhang, 2011, *B.flavus* Chen & Gong, **sp. n.** and *B.guttatus* Chen & Gong, **sp. n.**, are described and illustrated from China. The generic characteristics are redefined and photographs of the new species are provided. A checklist and a key to species of *Bambusicaliscelis* are also given.

## Introduction

The planthopper family Caliscelidae Amyot & Serville, 1843, including two subfamilies, five tribes, 76 genera, and more than 200 species (Bourgoin 2018), is a small group that widely distributed in the world. So far, in China, the taxa of the family contained four tribes (Caliscelini, Peltonotellini, Ommatidiotini and Augilini), 12 genera, and 29 species (Emeljanov 2008; Che et al. 2009, 2011; Chen et al. 2014; Meng et al. 2015). Two species of *Bambusicaliscelis* (*B.dentis* and *B.fanjingensis*), one species of *Pseudosymplanella* (*P.nigrifasciata*), one species of *Augilodes* (*A.binghami*), three species of *Symplana* (*S.brevistrata*, *S.lii* and *S.longicephala*), and five species of *Symplanella* (*S.brevicephala*, *S.hainanensis*, *S.recurvata*, *S.unipuncta* and *S.zhongtua*) were found on bamboo from China (Che et al. 2009; Chen and Zhang 2011; Chen et al. 2014; Yang and Chen 2014). Unfortunately, no more other information on host plants is available except for *S.recurvata* collected on *Neosinocalamus* sp.

The planthopper genus *Bambusicaliscelis* was established by Chen and Zhang (2011) based on two species, *B.dentis* and *B.fanjingensis*, from China, and placed in the tribe Caliscelini of the subfamily Caliscelinae (Hemiptera: Fulgoroidea: Caliscelidae). The two species of *Bambusicaliscelis* are similar but can be easily distinguished from each other by their male genitalia.

In this paper, two new species, *Bambusicaliscelisflavus* sp. n. and *Bambusicaliscelisguttatus* sp. n., were collected from bamboo. Their descriptions and illustrations are given. The generic characteristics are redefined. A checklist and a key to species of *Bambusicaliscelis* are given.

## Materials and methods

Terminology follows Chan and Yang (1994) and Chen and Zhang (2011). Dry specimens were used for the descriptions and illustrations. External morphology was observed under a stereoscopic microscope and characters were measured with an ocular micrometer. Measurements were given in millimeters; body length was measured from the apex of the head to tip of the abdomen in repose. The genital segments of the examined specimens were macerated in 10% NaOH, washed in water, and transferred to glycerin. Illustrations of the specimens were made with a Leica MZ 12.5 stereomicroscope. Photographs were taken with KEYENCE VHX-1000 system. Illustrations were scanned with CanoScan LiDE 200 and imported into Adobe Photoshop CS7 for labelling and plate composition.

The type specimens and material examined are deposited in the Institute of Entomology, Guizhou University, Guiyang, China (**IEGU**).

## Taxonomy

### Bambusicaliscelis

Taxon classificationAnimaliaHemipteraCaliscelidae

Chen & Zhang, 2011

Figs 1–12 , 13–24

Bambusicaliscelis Chen & Zhang, 2011: 95; Chen et al. 2014: 157.

#### Type species.

*Bambusicaliscelisfanjingensis* Chen & Zhang, 2011, by original designation.

#### Diagnosis.

General color yellowish brown to blackish brown. Vertex from apex to tip of abdomen with a pale longitudinal stripe along median line. Vertex with disc slightly concave, lateral margins subparallel, width at base wider than length in middle line. Frons rather broad, widest part under level of lower margin of eyes, length in median line longer than width; lateral margins distinctly carinate, from apex to level of lower margin of eyes subparallel then gradually incurved to frontoclypeal suture; median carina present, weak; submedian carinae arising from basal margin of frons, slightly divergent then convergent apically, not reaching to frontoclypeal suture; each lateral area between submedian carina and lateral carina with two rows include 12 small pustules. Postclypeus with median carina distinct, lateral carinate obscure. Rostrum reaching posterior trochanters. Pronotum broad transversely, 3-carinate, median carina weak, length in median line slightly shorter than vertex. Mesonotum 3-carinate, median carina weak, length in median line shorter than vertex and pronotum combined. Forewing with length slightly longer than width, anterior and posterior margins subparallel, apical margin subtruncate, veins obscure. Hindwing absent. Legs with fore and middle femora and tibiae normal. Hind tibiae with one spine at middle. Spinal formula of hind leg 6–3–2.

*Male genitalia.* Anal segment short, in dorsal view with length in middle line longer than broad at widest part. Pygofer in lateral view with ventral margin distinctly longer than dorsal margin, in posterior view long oval, with opening longer than broad. Aedeagus with phallobase tubular; phallus paired, slender and long, encircled in phallobase, tapering apically. Genital style broad, with a strong finger-like process apically arising from dorsal margin, directed basally.

#### Distribution.

China (Guizhou, Yunnan, and Guangxi).

#### Host plant.

Bamboo.

#### Checklist of species of *Bambusicaliscelis* Chen & Zhang

*B.dentis* Chen & Zhang, 2011; China (Guizhou).

*B.fanjingensis* Chen & Zhang, 2011; China (Guizhou).

*B.flavus* Chen & Gong, sp. n.; China (Yunnan).

*B.guttatus* Chen & Gong, sp. n.; China (Guangxi).

## Key to species of genus *Bambusicaliscelis*

**Table:** 

1	Vertex with anterior margin slightly convex (Figure 3); forewing yellow (Figs 1–2)	***B.flavus* sp. n.**
–	Vertex with anterior margin truncated; forewing yellowish brown to blackish brown	**2**
2	Phallus of male with 2–3 teeth-like processes (Chen and Zhang 2011: Figs 19–20)	*** B. dentis ***
–	Phallus of male without any teeth-like processes	**3**
3	Pygofer of male in posterior view ventral margin with medioventral process single (Chen and Zhang 2011: Figure 7)	*** B. fanjingensis ***
–	Pygofer of male in posterior view ventral margin with medioventral processes pair (Figure 21)	***B.guttatus* sp. n.**

## 

### Bambusicaliscelis flavus

Taxon classificationAnimaliaHemipteraCaliscelidae

Chen & Gong sp. n.

http://zoobank.org/2AE83E54-7C91-466C-AB23-654782A6FCEC

Figs 1–12

#### Measurements.

Body length (from apex of vertex to tip of abdomen): male 4.2–4.3 mm (N = 2); forewing length: male 1.7–1.8 mm (N = 2).

#### Description.

**Figures 1–12.**
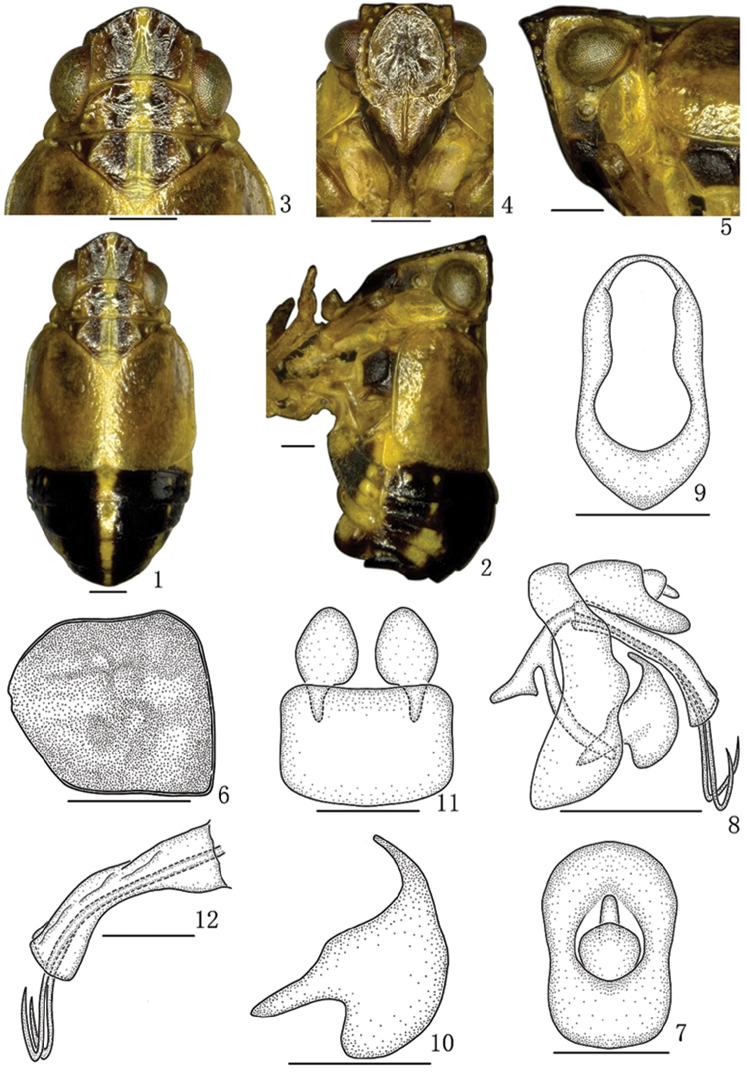
*B.flavus* sp. n., male **1** Male habitus, dorsal view **2** Male habitus, lateral view **3** Head and thorax, dorsal view **4** Face **5** Head and thorax, lateral view **6** Forewing **7** Anal segment, dorsal view **8** Male genitalia, lateral view **9** Pygofer, posterior view **10** Genital Styles, lateral view **11** Pygofer and genital styles, ventral view **12** Aedeagus, lateral view. Scale bars: 0.5 mm (**1–5, 7, 10–12**), 1 mm (**6, 8–9**).

*Coloration.* Body mainly yellowish brown. The longitudinal stripe from apex of vertex to tip of abdomen pale yellow, abdomen blackish brown (Figs 1–2). Frons (Figure 4) brown with the small yellowish white pustules between lateral and submedian carinae. Clypeus, antennae and legs yellowish brown. Eyes brown. Pustules of pro- and mesonotum (Figure 3) yellowish white. Forewing (Figs 1–2, 6) yellow.

*Head and thorax.* Vertex with anterior margin slightly convex, width of vertex (Figure 3) including eyes 0.9 times narrower than pronotum. Vertex (Figure 3) with length in middle line 0.7 times than width at base. Frons (Figure 4) 1.1 times longer in middle line than widest part, submedian carinae slightly keeled; areas between submedian carinae and lateral carinae slightly depressed. Pronotum (Figure 3) shorter in middle line than vertex (1:1.3). Mesonotum (Figure 3) 0.8 times as long as vertex and pronotum together in middle line. Forewing (Figure 6) with length 1.1 times than broad at widest part, veins obscure.

*Male genitalia.* Anal segment in dorsal view (Figure 7) with length 1.5 times longer in middle line than widest part, lateral margins slight concave; in lateral view (Figure 8) dorsal margin slightly convex, broadening apically, to apical 1/2 widest, thence abruptly narrowed, ventral margin slightly concave. Pygofer in lateral view (Figure 8) with posterior margin sinuate; in posterior view (Figure 9) nearly oval, with length 1.9 times than widest part; in ventral view (Figure 11) with posterior margin slightly concave, anterior margin slightly convex, two lateral margins subparallel. Genital style in lateral view (Figure 10) with median portion broad, large, apical margin slightly concave, with length 1.7 times as long as widest part; in ventral view (Figure 11) pear-like. Aedeagus with phallobase relatively large, truncate; phallus (Figs 8, 12) tubular, slender and long, tapering apically, apical 1/2 beyond apical margin of phallobase, then apical 1/4 dorsally reflexed.

#### Type material.

Holotype: ♂, **China**: Yunnan Province, Lushui County, Pianma Town (26°10'N, 98°38'E), 17 August 2008, Xiang-Sheng Chen; paratypes: ♂, data same as holotype.

#### Host plant.

Bamboo.

#### Distribution.

China (Yunnan).

#### Etymology.

The specific name is derived from the Latin words “*flavus*” which refer to its forewing color.

#### Differential diagnosis.

This new species is similar to *B.fanjingensis*, but differs in: 1) forewing yellow (dark brown in *fanjingensis*); 2) pygofer in posterior view, ventral margin without medioventral process (with a medioventral process in *fanjingensis*); 3) pygofer in lateral view with dorsal margin roundly convex and posterior margin sinuate (dorsal and posterior margin concave at middle in *fanjingensis*).

### Bambusicaliscelis guttatus

Taxon classificationAnimaliaHemipteraCaliscelidae

Chen & Gong sp. n.

http://zoobank.org/89A104FC-DFF4-4AFD-BBB2-6A62C9CE79DB

Figs 13–24

#### Measurements.

Body length (from apex of vertex to tip of abdomen): male 4.2 mm (N = 1); forewing length: male 1.7 mm (N = 1).

#### Description.

**Figures 13–24.**
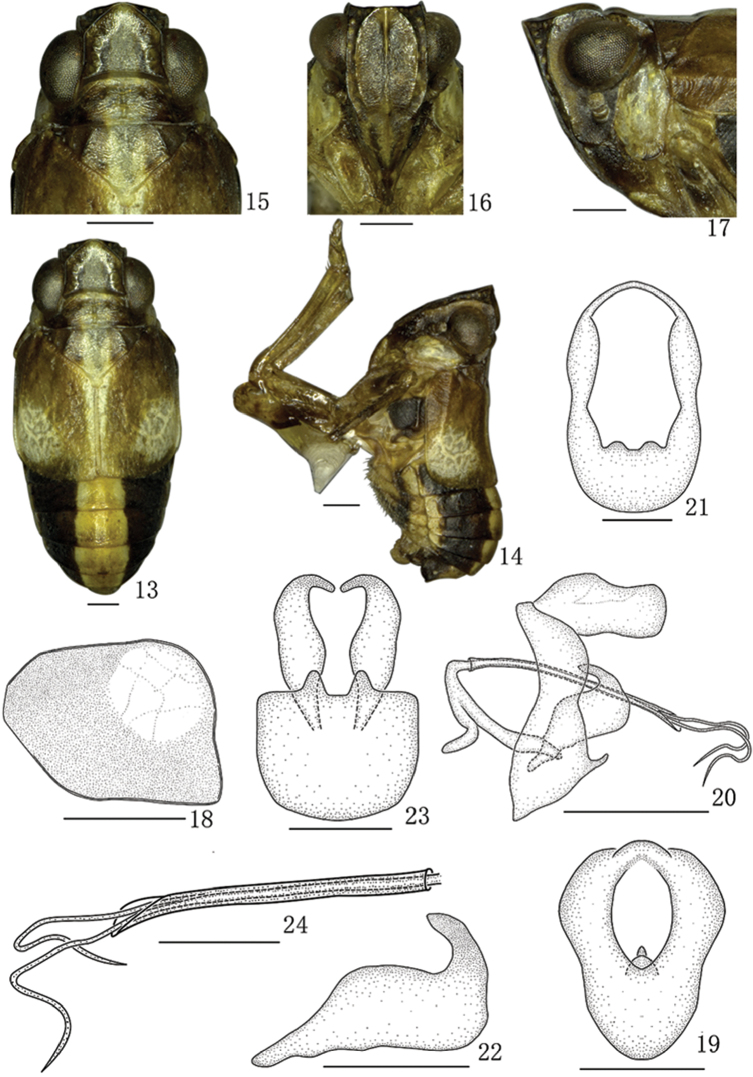
*B.guttatus* sp. n., male **13** Male habitus, dorsal view **14** Male habitus, lateral view **15** Head and thorax, dorsal view **16** Face **17** Head and thorax, lateral view **18** Forewing **19** Anal segment, dorsal view **20** Male genitalia, lateral view **21** Pygofer, posterior view **22** Genital Styles, lateral view **23** Pygofer and genital styles, ventral view **24** Aedeagus, lateral view. Scale bars: 0.5 mm (**13–17, 19, 21–24**), 1 mm (**18, 20**).

*Coloration.* Body mainly yellowish brown to blackish brown. The longitudinal stripe from apex of vertex to tip of abdomen pale yellowish white (Figure 13). Frons (Figure 16) dark brown with the small pustules yellowish brown between lateral and submedian carinae. Clypeus brown. Eyes and antennae dark brown. Forewing (Figs 13–14, 18) brown with one large yellowish white marking near apical margin. Legs brown.

*Head and thorax.* Vertex with anterior margin subtruncated, width of vertex (Figure 15) including eyes as long as pronotum. Vertex (Figure 15) with length in middle line 0.8 times than width at base. Frons (Figure 16) 1.3 times longer in middle line than widest part, submedian carinae slightly keeled, areas between submedian carinae and lateral carinae slightly depress. Pronotum (Figure 15) shorter in middle line than vertex (1:1.6). Mesonotum (Figure 15) 0.7 times as long as vertex and pronotum together in middle line. Forewing (Figure 18) with length 1.3 times than broad at widest part, veins obscure.

*Male genitalia.* Anal segment in dorsal view (Figure 19) with length 1.3 times longer in middle line than widest part, two lateral margins concave; in lateral view (Figure 20) dorsal margin slightly convex, the widest at apical 1/2, thence constricted, ventral margin slightly concave in the middle. Pygofer in lateral view (Figure 20) with posterior margin with upper half roundly convex, lower half truncated; in posterior view (Figure 21) nearly oval, with length 1.7 times as long as widest part; in ventral view (Figure 23) with posterior margin with two stout and short medioventral processes, anterior margin slightly convex, lateral margins subparallel. Genital style in lateral view (Figure 22) with basal 1/2 basally narrowing, median portion widest, apical margin slightly concave, with length 3.1 times as long as widest part, a strong finger-like process apically arising from dorsal margin, directed basad; in ventral view (Figure 23) long and narrow, with apex inward bent, nearly hook-like. Aedeagus with phallobase (Figs 20, 24) slender, long and tubular. Phallus (Figs 20, 24) tubular, much slender and longer, tapering apically, apical 1/2 beyond apical margin of phallobase, then apical 1/4 distinctly bent.

#### Type material.

Holotype: ♂, **China**: Guangxi, Damingshan National Natural Reserve (23°54'N, 108°37'E), 10 August 2011, Zai-Hua Yang.

#### Host plant.

Bamboo.

#### Distribution.

China (Guangxi).

#### Etymology.

The specific name is derived from the Latin words “*guttatus*” which refer to its forewing with a large yellowish white marking.

#### Differential diagnosis.

*B.guttatus* sp. n. is similar to *B.fanjingensis*, but differs in: 1) forewing brown with one large yellowish white marking (blackish brown, without any marking in *fanjingensis*); 2) pygofer in posterior view, ventral margin with two medioventral processes (medioventral process single in *fanjingensis*); 3) genital style in lateral view with dorsal process located apically, large and apical margin roundly convex (dorsal process located near apex, relatively slender and apex sharp in *fanjingensis*).

## Discussion

The *Bambusicaliscelis* Chen & Zhang, 2011 and *Thaiscelis* Gnezdilov, 2015 are readily distinguished from other known genera of Caliscelini by carination of the frons (Figs 4, 16; Gnezdilov 2015: figs 6–7). The genus differs from *Thaiscelis* in general coloration being yellowish brown to blackish brown (dark brown or black in *Thaiscelis*); vertex with anterior margin truncate or roundly convex (anterior margin acutely angulate in *Thaiscelis*); each side of frons between lateral margin and submedian carina with two rows include 12 small pustules (eleven small pustules in *Thaiscelis*).

*Bambusicaliscelis* may be seen as one of the most primitive members of tribe Caliscelini according to its “closed-tube” type of phallobase (Figs 12, 24), which is possibly the primitive (ancestral) condition compared to the “open-tube” type of other Caliscelini (Gnezdilov and Bourgoin 2009: figs 63–65) and Peltonotellini (Emeljanov 2008: figs 2–3), which may be treated as a derived condition.

## Supplementary Material

XML Treatment for Bambusicaliscelis

XML Treatment for Bambusicaliscelis flavus

XML Treatment for Bambusicaliscelis guttatus
